# Metabolite fingerprinting of phytoconstituents from *Fritillaria cirrhosa* D. Don and molecular docking analysis of bioactive peonidin with microbial drug target proteins

**DOI:** 10.1038/s41598-022-10796-7

**Published:** 2022-05-04

**Authors:** Basharat Ahmad Bhat, Wajahat Rashid Mir, Bashir Ahmad Sheikh, Mustafa Alkanani, Manzoor Ahmad Mir

**Affiliations:** 1 Department of Bioresources, School of Biological Sciences, University of Kashmir, Srinagar, 190006 India; 2 College of Applied Medical Sciences, Almaarefa University, Riyadh, 11597 Kingdom of Saudi Arabia

**Keywords:** Drug discovery, Immunology, Microbiology, Diseases

## Abstract

*Fritillaria cirrhosa* D. Don (Liliaceae), a valuable and critically endangered medicinal herb of northwest India, including Jammu and Kashmir, grows in temperate to alpine regions of the Himalaya. It is known as the traditional herb for cardiovascular diseases, respiratory diseases, and metabolic disorders. The plant bulbs are precious and are used to cure many other health complications. The current study analysed the phytoconstituents by liquid chromatography-mass spectrometry (LC–MS) of different crude extracts (methanolic, petroleum ether, and ethyl acetate) of *F. cirrhosa*. The LC–MS analysis from the bulbs of *F. cirrhosa* yielded 88 bioactive compounds, with the vast majority having therapeutic applications. Further, determination of minimum inhibitory concentrations (MICs) by broth microdilution method of *F. cirrhosa* against tested bacterial and fungal pathogens showed remarkable results with MICs ranging between 6.25–200 µg/mL and 50–400 µg/mL, respectively. Subsequently, these 88 identified phytocompounds were tested for their bioactivity through ADMET prediction by SwissADME and in silico molecular docking studies. Results revealed that Peonidin might have maximum antibacterial and antifungal activity against various microbial protein drug targets among the phytochemical compounds identified. Furthermore, the highest binding affinity complex was subjected to molecular dynamic simulation (MDS) analysis using Desmond Schrodinger v3.8. The root-mean-square deviation (RMSD) graphs obtained through the molecular dynamic simulations indicated the true bonding interactions, further validated using the root-mean-square fluctuation (RMSF) graphs which provided a better understanding of the amino acids present in the proteins responsible for the molecular motions and fluctuations. To our best knowledge, this is the first description of the phytochemical constituents of the bulbs of *F.cirrhosa* analyzed through LC–MS, which show pharmacological significance. The in silico molecular docking and molecular dynamics study of peonidin was also performed to confirm its broad-spectrum activities based on the binding interactions with the antibacterial and antifungal target proteins. The present study results will create a way for the invention of herbal medicines for several ailments by using *F. cirrhosa* plants, which may lead to the development of novel drugs.

## Introduction

Medicinal plants have played a pivotal role in primary health care and offer a rich source of novel bioactive compounds in drug discovery and development^1,2^. Infectious diseases are public health problems and a significant cause of death worldwide^3–5^. Infections due to pathogenic microorganisms cause a severe concern to human health^6^. Increasing cases of drug resistance, unwanted side effects of existing antibiotics, and the reappearance of earlier known infections have demanded the need for new, safe and effective antimicrobial agents^3,7–10^. In drug development, virtual screening like drug-likeness and ADMET analysis are computational methods to find compounds that are likely to exhibit physiological activity in a short time and at a low cost using various in silico simulation methods^11^.

The *Liliaceae* family harbours a yellow Himalayan Fritillary^12–14^ that flowers yellowish to green or brownish to purple bell-shaped flowers commonly found in the alpine regions ranging from 2700–4900 m^13,14^, and are native to China and Indian subcontinent regions. It is widely known as *Fritillaria cirrhosa* D. Don*.* is a perennial and critically endangered medicinal herb of the Astavarga group [a group of eight medicinal herbs used in traditional medical knowledge (TMK) and the traditional medical system (TMS) in India]. Grows in open sunny slopes of temperate to alpine regions of the Himalaya^15^. It occurs in western temperate Himalaya from Kashmir to Kumaon from 2700 to 4035 m asl^16^ and from Pakistan to Uttarakhand at 2700–4000 masl^17^. As per the field survey conducted during the study, the species was found in the Gulmarg, Kongdori, Khillenmarg and Botapathri regions of Kashmir. According to the records of the KASH (Kashmir University Herbarium), the species has been found in the Gulmarg, Apharvat, Thajwas, Zojila, Langait, and Gurez regions of Kashmir. The bulbs of this plant are rich in bioactive compounds like sipeimine, which is used to treat many respiratory disorders. More than a hundred important bioactive phytocompounds and other significant chemical compounds have identified, isolated and extracted till date from *F. cirrhosa*^18^*.* These components include alkaloids like peiminine, peimine, peimisine, imperialine etc.^19–21^. Chinese medicinal practitioners have employed this plant to implicate its medicinal and therapeutic potency^22^. It's a bitter tonic and gastrointestinal stimulant that relieves fevers and urinary tract infections and is a medicine for 80 diseases^23^. It's used as a refrigerant, diuretic, galactagogue, expectorant, and aphrodisiac, among other things. It's used to treat rheumatism, tuberculosis, and haematemesis. It also relieves discomfort in pregnant women, encourages the growth of flesh, and relieves various aches and symptoms^24^. The alkaloids found in the bulbs of *F. cirrhosa* have been shown to have hypotensive^21,25^, anti-inflammatory^21,26^, antitumor^21,25,27^, antitussive, expectorant^21,26,28^, and antiasthmatic activities^21,28^.

These findings support the hypothesis that *F. cirrhosa* is a valuable medicinal plant. Although the extracts of *F. cirrhosa* were traditionally utilized and therefore extensively studied and reported for various pharmacological activities. Since the chemistry and antimicrobial studies of the *F. cirrhosa* growing in Jammu & Kashmir seems not to have been worked out, we carried out the present studies to characterize the various extracts and investigate its antimicrobial activity through molecular docking studies, ADMET analysis and in vitro approaches for the first time. Herein, we report the antimicrobial activity of three different extracts against various bacterial and fungal strains.

## Methodology

### Collection of plant material

*F. cirrhosa* bulbs were collected from four locations (viz Razdhan top, Sadhna Pass, Tangdhar Main, Apharvat Gulmarg) in 2020 of Jammu and Kashmir. Geographical coordinates of the site sample are mentioned in (Table 1). The plant was identified by Akhtar Malik, Curator and Taxonomist at the University of Kashmir's Department of Botany.

**Table 1:** Details of the collection sites of bulb samples of *F. cirrhosa* from five significant sites in Jammu and Kashmir union territory, India, for carrying ethnopharmacological studies.

Sampling site name	Geographical coordinates			Date of sample collection	Sample type
	Latitude	Longitude	Altitude (AMSL)		
Razdhan top	34° 37′ 59.88″ N	74° 49′ 59.88″ E	3545	26-05-2020	Bulb
Sadhna Pass	34° 24′ 05.8″ N	73° 57′ 11.8″ E	10,020	12-06-2020	Bulb
Tangdhar Main	34° 23′ 53.0″ N	73° 51′ 30.9″ E	1820	30-06-2020	Bulb
Apharvat Gulmarg	33° 59′ 58″ N	74° 19′ 32″ E	4390	26-06-2020	Bulb

### Preparation of extracts

Petroleum ether, ethyl acetate, and methanol were chosen as extraction solvents based on their polarity index. Finely ground powdered plant material (5 g) was extracted in the solvents as mentioned above. To maintain the natural antimicrobial activity in a sample extract and allow maximum extraction, the mixture was stored on an incubator rotatory shaker at (200 rpm) 25 °C for 48 h. After filtering the extracts through Whatman filter paper (No. 1), the filtrate was centrifuged for 15 min at 8000 rpm at 12 °C to obtain a clear supernatant. The extracts produced in various solvents were evaporated under reduced pressure in a rotatory evaporator and diluted to obtain a stock solution with a concentration of 10 mg/mL, which was stored in falcon tubes coated in aluminium foil at 4 °C in the refrigerator for later use^29^.

### Liquid chromatography–mass spectrometry (LC/MS)

Nexera UHPLC with a quaternary pump, central degassing unit, Autosampler, and DAD unit was used for the LC–MS analysis. Elution was carried out at a rate of 1 mL/min with petroleum ether, ethyl acetate and methanol as solvents. After ultrasonication, all solvents were passed through 0.45 µm nylon paper. At 270 nm, the chromatograms were examined, and the findings were obtained using lab-developed software^30^. The plant extracts were formulated using petroleum ether, ethyl acetate, methanolic, and bioanalysis to identify important metabolites and phytocompounds. The presence of 88 phytocompounds were ascertained by executing LC–MS analysis on these extracts.

### Bacterial strain and culture preparation

The microbes were procured from IMTECH's Gene Bank in Chandigarh and American Gene Bank. For antibacterial and antifungal activity, six bacterium strains and three fungus strains were investigated (*Escherichia coli* MTCC443, *Klebsiella pneumonia* MTCC19, *Microbacterium luteus*, *Streptococcus pneumonia* MTCC 655, *Haemophilus influenzae* MTCC 3826, *Neisseria mucosa* MTCC 1722 and *Candida albicans* ATCC 24433*, Candida glabrata* ATCC2001*, Candida Parapsilosis* ATCC90018).

All the cultures were maintained at 37 °C for better growth in the incubator. For each experiment, the prepared aliquots were thawed and sub-cultured in broth with 10% ADC (albumin-dextrose-catalase Himedia, India), 0.2% Glycerol (Merck, India), and 0.05% Tween 80 (Merck, India) and cultures were grown till stationary phase and further processed for other experiments.

### Minimum inhibitory concentration by broth dilution method

Minimum inhibitory concentration in the present study was performed through Muller Hinton broth dilution method. In the present study, 10% ADC was supplemented in broth followed by performing a two-fold serial concentration from 0.00 to 0.20 µg/mL of antibiotics, including ciprofloxacin and amphoterecin-B in different combinations with fungal and bacterial cultures. Moreover, each inoculated plate was kept in an incubator at 37 °C for 24 to 48 h. To monitor the growth of cells and observe growth inhibition, the minimum inhibitory concentration of the drug was noted. MIC was considered as the lowest concentration of an antimicrobial at which no balanced growth was observed. In addition, colony-forming units were, measured on day 28th of incubation at 37 °C for better use.

In terms of drug interaction, the microbial cultures were grown in various concentrations of ciprofloxacin and amphotericin B in multiple combinations. The antimicrobials were added to fabricate 8 × MIC for both the antimicrobials followed by two fold dilutions leading to 1/64 × MIC concentration ranges of each drug both alone and in combination were assured^31^.

### Ligand and receptor preparations

The molecular docking analysis was carried out for all the selected phytocompounds from the LC–MS data fractions. Chemical structures of phytocompounds retrieved from PubChem database in SDF format and converted into protein data bank (PDB) format using PyMol Version 3.0. Software. The selected targets for this study were based on previous studies, and all the 3D crystal structures were fetched from (PDB) in PDB format. The structural information of the Peonidin identified from the *Fritillaria* *cirrhosa* was also retrieved from the PubChem database in SDF format and converted into PDB format using an open babel server. Peonidin is one of the compounds identified from LC–MS analysis. It exhibits apoptotic effects in the breast cancer cells^32^.

Peonidin is an anthocyanide that can be easily eliminated from the human body. Anthocyanins (ACNs), including peonidin, are natural bioactive compounds with many pharmacological effects: antioxidant, anti-inflammatory, prevention of age-related chronic diseases: cardiovascular disease (CVD), cancers, neurodegenerative, and eye-related diseases. ACNs also have antiviral properties. Recent in vitro studies have shown that they can inhibit the replication of viruses such as herpes simplex, parainfluenza virus, syncytial virus, HIV, rotavirus, and adenovirus^33^. The broad spectrum of pharmacological properties supported by preclinical and clinical evidence associated with low toxicity make their pharmacotherapeutic use very attractive. Peonidin was selected as an appropriate ligand in the current study based on its pharmacological properties. Compounds with antimicrobial activities disable bacteria by targeting key bacterial metabolism components, including dihydropterote synthase (DHPS), penicillin binding protein (PBP), elongation factor Tu, 1,3 beta glycan, ABC transporter and beta-tubulin . These six key proteins play a critical role in the life cycle of fungi and bacteria. Penicillin-binding proteins (PBPs) are membrane-associated proteins that play a vital role in forming cell wall^34,35^. As previously indicated, antibiotics target the dihydropteroate synthase enzyme^36^. This enzyme participates in the folate synthesis pathway, directly connected to nucleic acid synthesis^37^. Inhibiting this enzyme with a strong antibiotic will have a more profound and irreversible effect on the bacterium's protein production. EF-Tu's primary role is to transport amino-acylated tRNAs to the ribosome. Since the 1970s, antibiotics have targeted EF-Tu as a therapeutic^38^. ABC transporters (ATP-binding cassette) are found in all three domains of life and mediate transmembrane transport of a wide range of substrates, including medicines, proteins, carbohydrates, amino acids, ions and carbohydrates. Glucans have long been known to have immunomodulatory effect^39^. Tubulin are the protein subunits of microtubules which helps in cell division of prokaryotic and eukaryotic organisms^40^. In view of this, the molecular docking study was carried out to examine the binding interactions of peonidin with these microbial drug targets. All the targeted receptors were fetched from the protein data bank (PDB) in the PDB format and subjected for purification and refinement. All the receptors were prepared for molecular docking analysis using Biovia Discovery Studio and eliminate undesirable ligands, molecules of water, and other impurities from all of the proteins involved in the study.

### Purification and refinement of proteins and ligands

**Figure 1:**
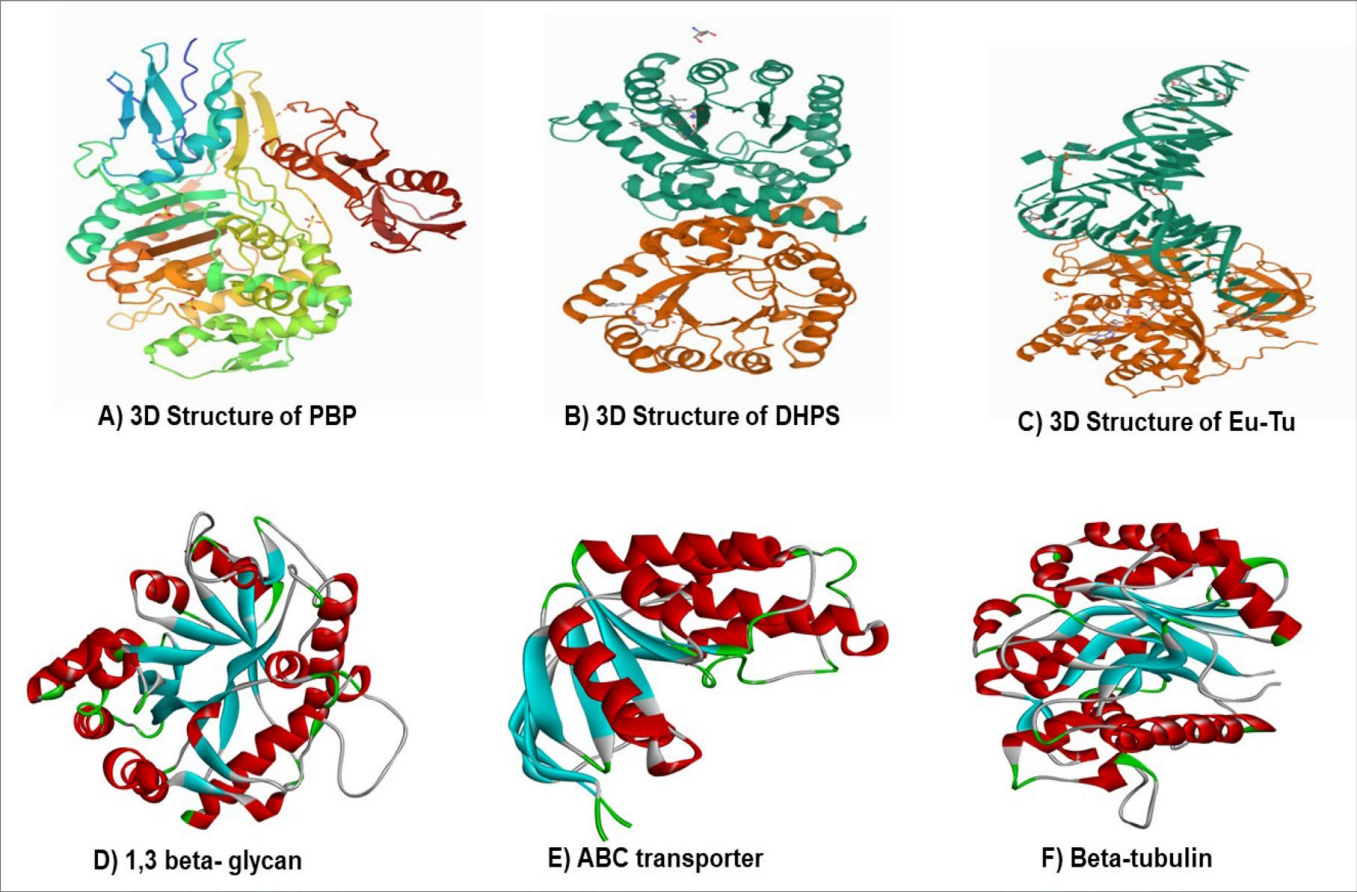
3D structure of different bacterial and fungal proteins.

Biovia Discovery studio was used to eliminate undesirable ligands, molecules of water, and other impurities from all of the proteins involved in the study. Polar hydrogens were added to the protein during the preparation process for improved interactions, followed by Kollman charges. The 2D structure and 3D structure of peonidin were retrieved using the PubChem database (PubChem CID: 5281708). The 3D structures of dihydropteroate synthase (DHPS), penicillin-binding protein (PBP), elongation factor Tu, 1,3 beta glycan, ABC transporter and beta-tubulin were retrieved from the Protein Data Bank (Fig. 1).

### Molecular docking studies

The docking calculation and algorithms to reproduce the pocket binding residues was verified employing co-crystal ligands. In the current study, we have used the Lamarckian Genetic Algorithm in Autodock Vina 4.2 to initiate the docking analysis with the standard parameters. Furthermore, all the transformation were clustered by considering the 4.0A RMSD and the most favourable binding poses was selected depending on the lowest free energy and inhibition constant. The molecular docking studies were performed on all selected phytocompounds from the LC–MS data sections. Chemical structures of phytocompounds were obtained in SDF format from the PubChem database and were translated to PDB format with PyMol Version 3.0. The targets for this investigation were chosen based on the evidence from past research, and all 3D crystal structures were collected in .pdb format from the Protein Data Bank. Based on the isolation modes and species-specific variants, the crystal structures of proteins such as dihydropterote synthase, penicillin-binding protein, and elongation factor Tu (Ef-Tu), 1,3 beta glycan, ABC transporter and beta-tubulin were retrieved.

### In-silico drug-likeness and toxicity predictions

Drug-likeness of compounds was predicted based on an already established concept by Lipinski et al.^41^. The structures of all some pharmacologically important compounds were converted to their canonical simplified molecular-input line-entry system (SMILE). They submitted to the SwissADME and PreADMET tool to estimate in silico pharmacokinetics, such as the number of hydrogen donors, hydrogen acceptors, rotatable bonds, and total polar surface area of a compound. These compound’s organ toxicities and toxicological endpoints were predicted using PreADMET and OSIRIS Property^42^. The selection of compounds as drug candidates was determined by a drug score parameter. The higher the drug score value, the higher the compound's chance of being considered a drug candidate^43^.

### Molecular dynamic simulation

The Desmond Schrodinger package was employed for the molecular dynamic simulation with the highest binding affinity docked complex to analyse the docked calculation and interpretation. The analysis indulged simulation run for 100 ns to check the stability of structures. Desmond's inbuilt parameters were also used for protein preparation and bond orders. In the case of bond orders, the addition of hydrogen binds, and filling of missing residues are also optimized during the initial process. OPLS force field was used to initiate the builder system for the standard simulation grid box. And the conformation were retrieved in the form of simulation trajectories which could be analyzed using Root mean square deviation and root mean square fluctuation graphs through hydrogen bond analysis and radius of gyration plots.

## Results

### Preliminary phytochemical screening

The phytochemical study of various extracts from the bulbs of *F. cirrhosa* revealed a variety of phytochemicals. The essential constituents present in petroleum ether, ethyl acetate and methanolic extracts as represented in Table 2.

**Table 2:** Phytochemical pre-screening of *F. cirrhosa*  bulb extracts. The marks ‘+’ and ‘−’ indicate ‘present’ and ‘absent’, respectively.

Tests	Inference extract	Petroleum ether	Ethyl acetate	Methanol
**Carbohydrates**				
Molish’s test	Violet ring	−	−	−
Fehling’s test	Brick red ppt	−	+	+
Benedict’s test	Red ppt	−	−	−
Selwinoff’s test	Pink colour	−	+	+
**Tannins**				
5% FeCl_3_	Yellow colour	−	+	+
Lead acetate	White ppt	−	−	−
**Saponins**				
Foam test	Foaming	+	+	+
Froth test	Frothing	+	+	+
**Flavonoids**				
Shinoda test	Pink colour	+	+	+
**Phenolics**				
1% FeCl_3_	Black-blue colour	+	+	+
**Anthraquinone glycosides**				
Borntrager’s test	Pink colour	−	−	−
**Terpenoids**				
Salkowski’s test	The golden yellow ring at the junction	+	+	+
**Alkaloids**				
Wagner’s test	Brown ppt	−	+	+
Dragendroff’s test	Orange ppt	−	+	−
Mayer’s test	Cream ppt	−	−	+
Wagner’s test	Yellow ppt	−	+	+

### Chemical composition

**Figure 2:**
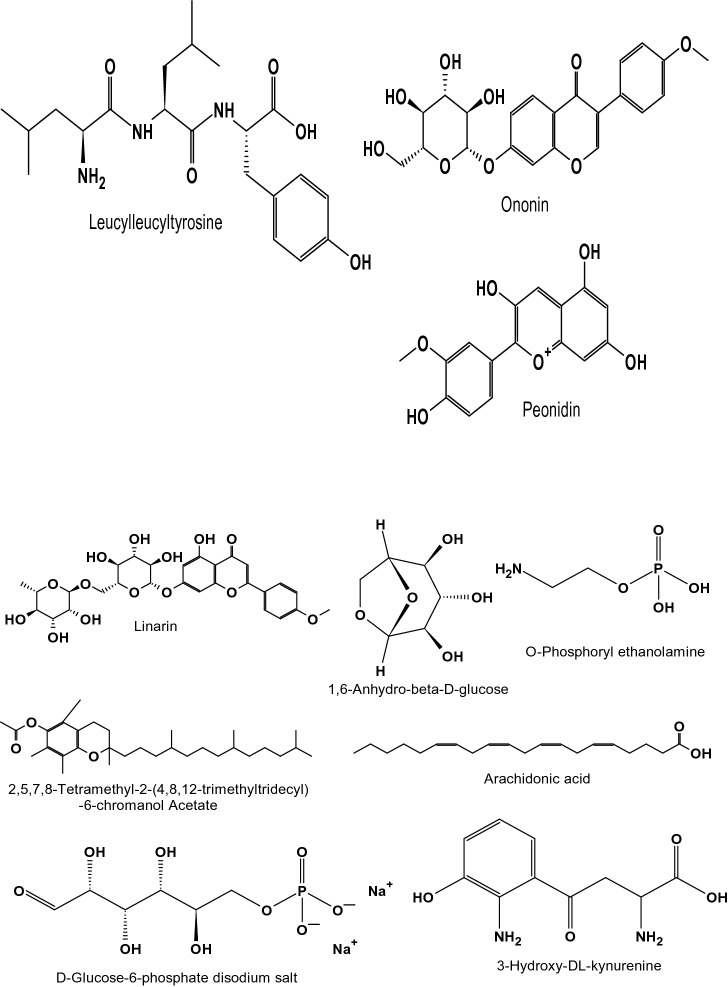
Structures of the compounds identified based on LC–MS from *F. cirrhosa* drawn by ChemDraw Pro 16.0 Suite (Perkin Elmer, USA).

**Figure 3:**
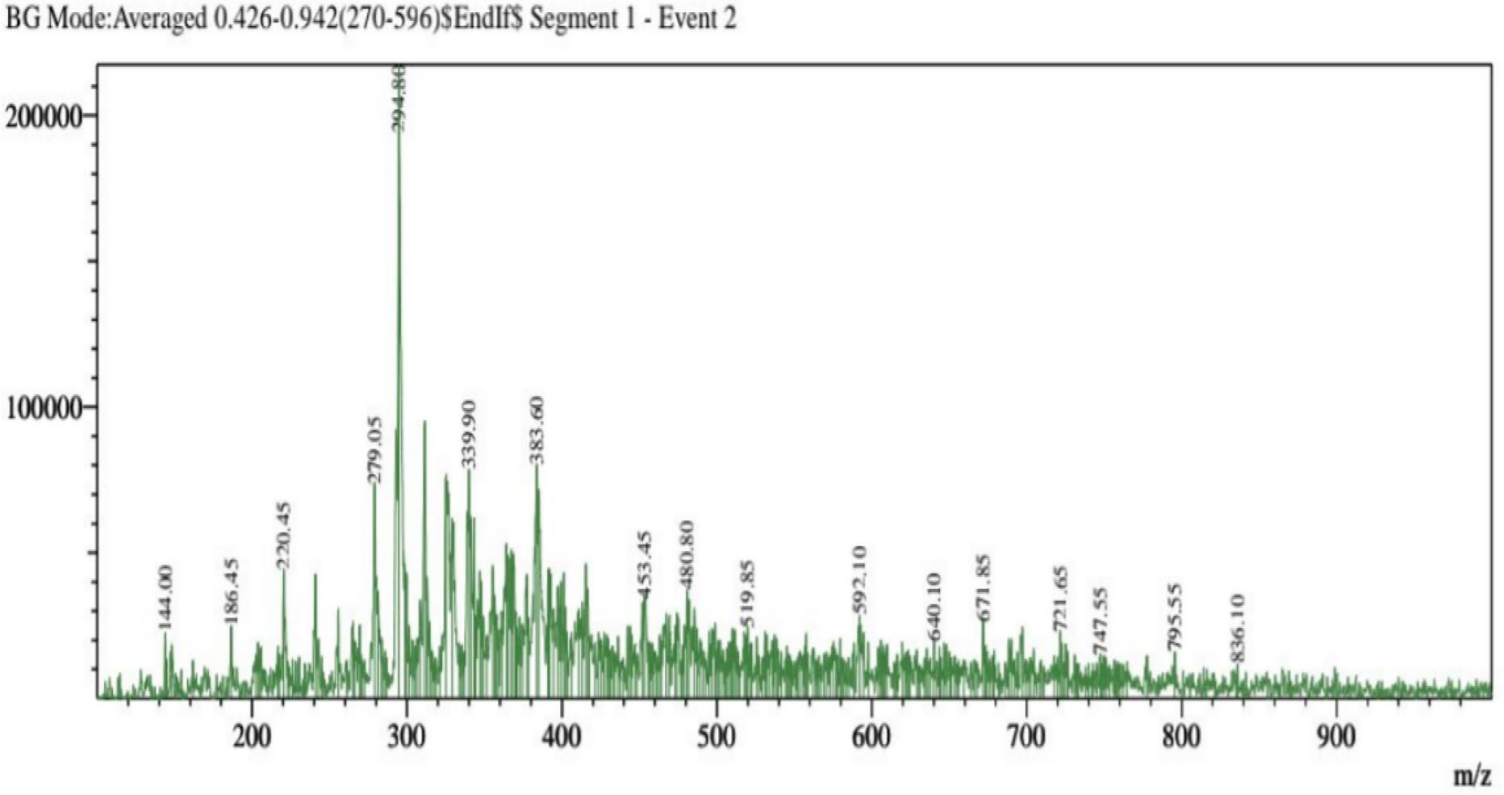
LC–MS-ESI–MS chromatograms of reference compounds using Nexera in Petroleum ether.

**Figure 4:**
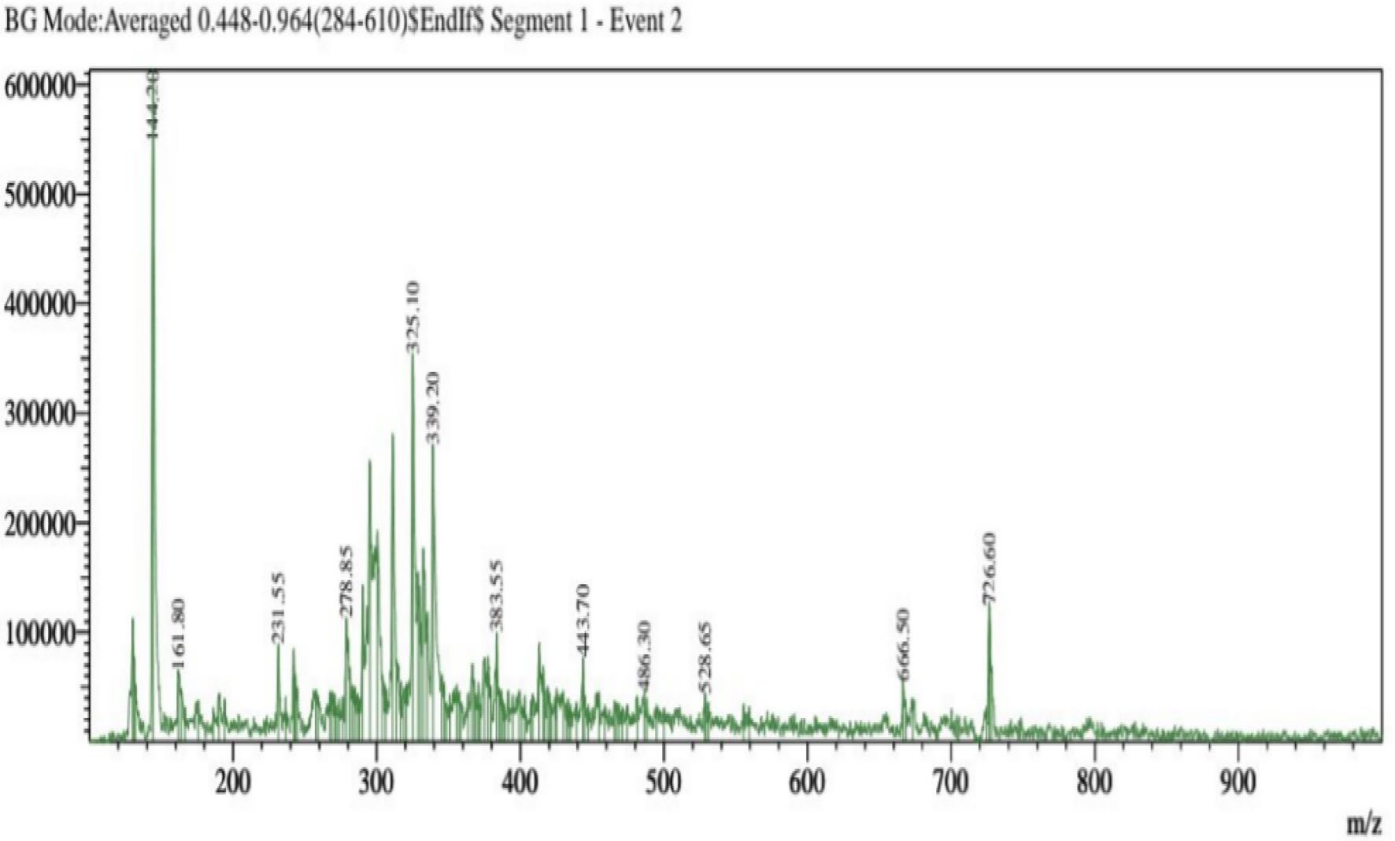
LC–MS-ESI–MS chromatograms of reference compounds using Nexera in Ethyl acetate.

**Figure 5:**
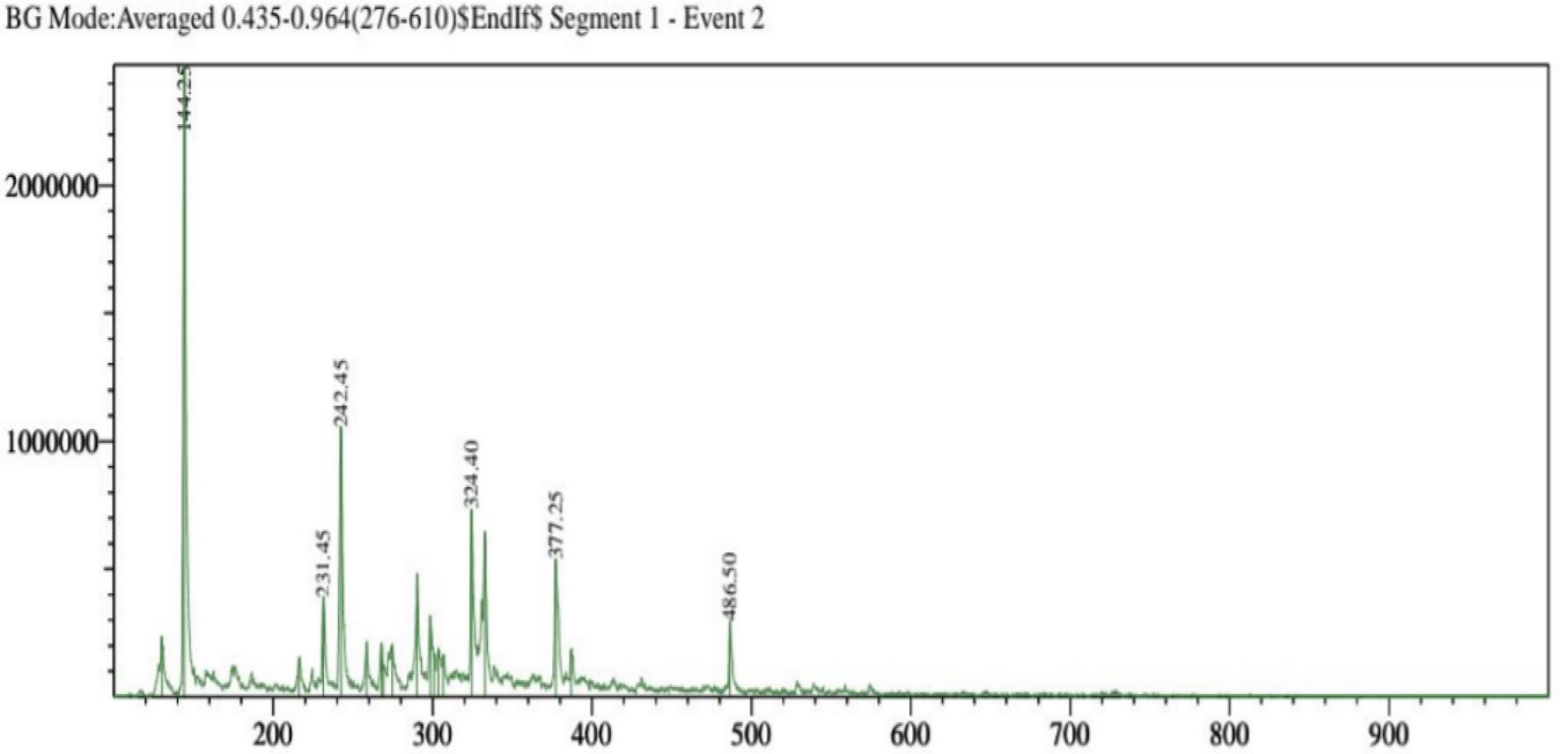
LC–MS-ESI–MS chromatograms of reference compounds using Nexera in Methanolic extract.

The separation of individual components presents in a solvent-based on their mass/charge ratio is the working principle behind Liquid Chromatography-Mass Spectroscopy (LC–MS). The chemical composition of the extracts from the *F. cirrhosa*, analyzed by LC–MS, are represented in (Table 3; Fig. 2). The LC–MS total ion chromatograms of different extracts of *F. cirrhosa* are shown in (Figs. 3, 4, 5). The solvent extraction carried out by Soxhlet extraction was subjugated to LC–MS analysis to obtain 88 important bioactive phytocompounds (Supplementary data file). A total of 22 phytocompounds was obtained using the methanolic extract etc. d-Glucose-6-phosphate disodium salt, 3-Hydroxy-dl-kynurenine, Leucylleucyltyrosine, and Ononin are a few compounds procured through the methanolic extract. Linarin, 1,6-Anhydro-beta-d-glucose, O-Phosphoryl ethanolamine were significant proportions among the 28 phytocompounds obtained through the petroleum ether extract. Peonidin, 2,5,7,8-Tetramethyl-2-(4,8,12-trimethyltridecyl)-6-chromanol Acetate, Arachidonic acid, and 19 other compounds were derived using the ethyl acetate extract.

**Table 3:** The major components found in *F. cirrhosa* based on LC–MS analysis.

Compound name	Molecular formula	Theoretical	Measured (g mol^−1^)
**Petroleum ether extract**			
Linarin	C_28_H_32_O_14_	592.179	591.54
1,6-Anhydro-beta-d-glucose	C_6_H_10_O_5_	162.14	161.045
O-Phosphoryl ethanolamine	C_2_H_8_NO_4_P	141.019	142.11
**Ethyl acetate extract**			
2,5,7,8-Tetramethyl-2-(4,8,12-trimethyltridecyl)-6-chromanol Acetate	C_31_H_52_O_3_	472.391	473.68
Arachidonic acid	C_20_H_32_	304.467	304.240234
**Methanolic extract**			
d-Glucose-6-phosphate disodium salt	C_6_H_11_Na_2_O_9_P	414.6	413.239
3-Hydroxy-dl-kynurenine	C_10_H_12_N_2_O_4_	145.16	144.04
Leucylleucyltyrosine	C_21_H_33_N_3_O_5_	430.126	431.4
Ononin	C_22_H_22_O_9_	430.381	431.64
Peonidin	C_16_H_13_O_6_	300.271	301.27

### Minimum inhibitory concentration

In terms of novel antimicrobial drugs, we have investigated the antimicrobial compounds extract from *F. cirrhosa* that showed significant inhibitory activity shown in Table 4 and observed to be non-toxic. The present study elaborates the detailed antimicrobial activity and in silico study for better validation.

**Table 4:** In vitro antimicrobial activity of different extracts of *F. cirrhosa.*

Strain	MIC (µg/mL)			
	PE	EA	ME	CIP/AMF-B
*Escherichia coli* (MTCC 443)	> 400	200	> 400	0.625
*Micrococcus luteus* (MTCC10240)	200	200	> 400	1.25
*Klebsiella pneumoniae* (ATCC 70063)	200	100	> 400	0.039
*Streptococcus pneumonia* (MTCC 655)	12.5	12.5	100	0.625
*Haemophilus influenzae* (MTCC 3826)	400	> 400	> 400	1.25
*Neisseria mucosa* (MTCC 1772)	50	6.25	400	0.312
*Candida albicans* (ATCC 24433)	> 400	400	> 400	1.25
*Candida glabrata* (ATCC2001)	> 400	> 400	> 400	2.5
*Candida Paropsilosis* (ATCCC90018)	> 400	> 400	> 400	2.5

CIP: Ciprofloxacin (reference antibacterial agent) and AMF-B: Amphotericin-B (Reference antifungal agent): PE: Petroleum ether; EA: Ethyl acetate: ME: Methanol.

In the present study, MICs of standard antimicrobial drug targets ciprofloxacin and amphotericin-B through broth dilution were 0.625, 1.25, 0.039, 0.625, 1.25, 3.12, 1.25, 2.5, and 2.5 µg/mL, respectively. The minimum inhibitory concentration of different plant extracts showed better outcomes than co-crystals shown in Table 4.

### Molecular docking analysis

**Figure 6:**
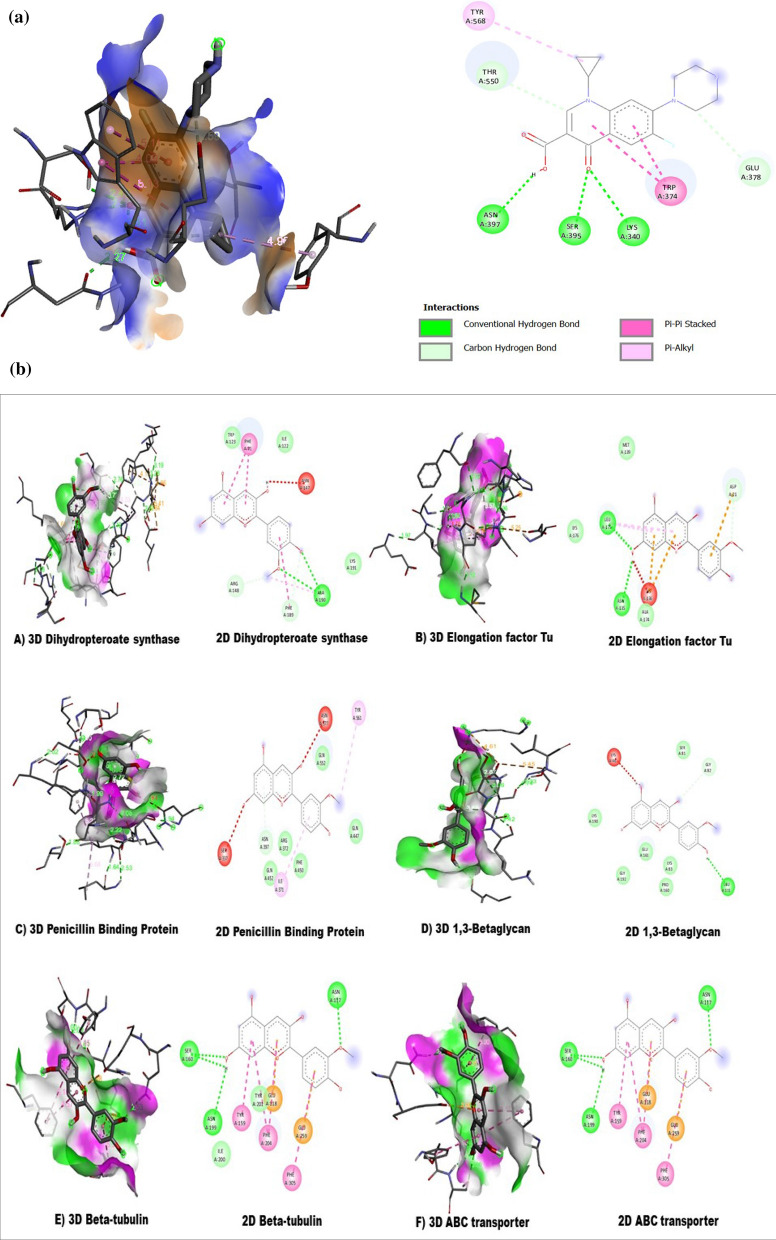
(**a**) The 2D and 3D intermolecular contact between ciprofloxacin and Pencillin binding protein. Chemical structures were drawn by ChemDraw Pro 16.0 Suite (PerkinElmer, USA) and analyzed by the Discovery studio visualizer (BIOVIA Discovery studio 2020 Client). (**b**) The 3D and 2D intermolecular contact between A) Dihydropteroate synthase B) Elongation factor Tu and C) Penicillin Binding Protein D) 1,3-Betaglycan E) Beta-tubulin F) ABC transporter with peonidin . Chemical structures were drawn by ChemDraw Pro 16.0 Suite (PerkinElmer, USA) and analyzed by the Discovery studio visualizer (BIOVIA Discovery studio 2020 Client).

The LC–MS analysis revealed that *F. cirrhosa* bulb extracts contained 88 bioactive compounds (Table 3). These phytocompounds were analyzed for activities against bacterial and fungal target proteins. The docking studies were carried out for phytoligands using the AutoDock Vina programme to elucidate the binding affinities to the target proteins. In general, among the 88 phytocompounds. All the six microbial drug targets related protein structures were docked against best-screened ligand peonidin obtained after ADME screening based on different parameters like solubility, toxicity, absorption, molecular weight and excretion. Docking was performed using AutoDock Vina with Chimera Plugin. Subsequently, the best-docked complex was screened based on their binding energy. Bonding between ligand and target protein were visualized using Biovia discovery studio. 2D-interaction diagrams showing different bonds formed between ligand and target protein were also visualized using Biovia discovery studio. Peonidin (2-(4-hydroxy-3-methoxyphenyl) chromenylium-3,5,7-triol) exhibited the best binding conformations with the lowest binding energy values with bacterial (− 8.2 kcal/mol), fungal (− 8.2 kcal/mol) target proteins (Tables 5 and 6). Our findings are supported by a previous study that showed that the lower the binding energy score, the better the protein–ligand binding stability was identified^44^. The peonidin formed the most excellent ligand–protein complexes with six tested microbial target proteins compared with other compounds. According to the binding energies, the docking results of peonidin with target proteins were ranked. The docking results of peonidin with the proteins, such as dihydropterote synthase, penicillin-binding protein, elongation factor Tu, 1, 3 beta glycan, ABC transporter and beta-tubulin confirmed that the ligand has a higher affinity for penicillin-binding protein, which is a key regulator in cell wall synthesis and maintenance (Fig. 6). In comparison to ciprofloxacin, the extracted peonidin natural molecule comparatively showed same docking efficiency with PBP within the binding pocket. The docked structure was imaged to illustrate the ligand interactions with significant amino acids TYR A:561, ILE A:371, ASN A:397, PHE A:450, and GLN A:452 through Vander Waal forces as well as hydrogen bonding (Fig. 6). The binding affinity of the selected compound against antibacterial and antifungal proteins are represented in (Tables 5, 6, 7, 8).

**Table 5:** The binding affinity of the selected compound against bacterial proteins.

Ligand	Dihydropteroate synthase (DHPS)	Penicillin binding protein	Elongation factor Tu (EF-Tu)
Peonidin	− 6.7 kcal/mol	− 8.2 kcal/mol	− 7.4 kcal/mol

**Table 6:** Binding affinity of selected compound against fungal receptors.

Ligand	1,3-Beta glycan	ABC transporter	Beta-tubulin
Peonidin	− 8.2 kcal/mol	− 7.9 kcal/mol	− 7.1 kcal/mol

**Table 7:** Binding affinity of ciprofloxacin compound against pencillin binding protein (PBP).

Complex	Dock score
Ciprofloxacin_(PBP)	− 8.4 kcal/mol

**Table 8:** Bond length and amino acids of docked compounds.

Ligand	Protein	Bond Length	Amino acids
Peonidin	Dihydropteroate synthase (DHPS)	3.78, 4.19, 2.19, 1.41, 4.66, 4.18, 2.86	TRP, PHE, ILE, ASN, ARG, ALA, LYS
Peonidin	Penicillin Binding Protein	1.11, 5.01, 3.86, 4.12, 3.98, 4.23	SER, ASN, GLN, TYR, ILE, PHE
Peonidin	Elongation factor Tu (EF-Tu)	2.18, 3.01, 4.12, 5.01, 3.87, 4.11	MET, ASP, LEU, LYS, ASN, ALA
Peonidin	1,3-Beta glycan	4.75, 2.53, 5.14, 4.95, 0.95, 5.34, 1.23	SER, ASN, TYR, ALU, PHE, ASN, ILE
Peonidin	ABC transporter	4.61, 5.45, 2.87, 2.12, 3.46	LYS, SER, GLY, LEU, PRO
Peonidin	Beta-tubulin	5.30, 3.78, 3.01, 2.34, 5.01, 2.11	SER, ASN, TYR, ALU, PHE, ASN

### Molecular dynamic simulation

**Figure 7:**
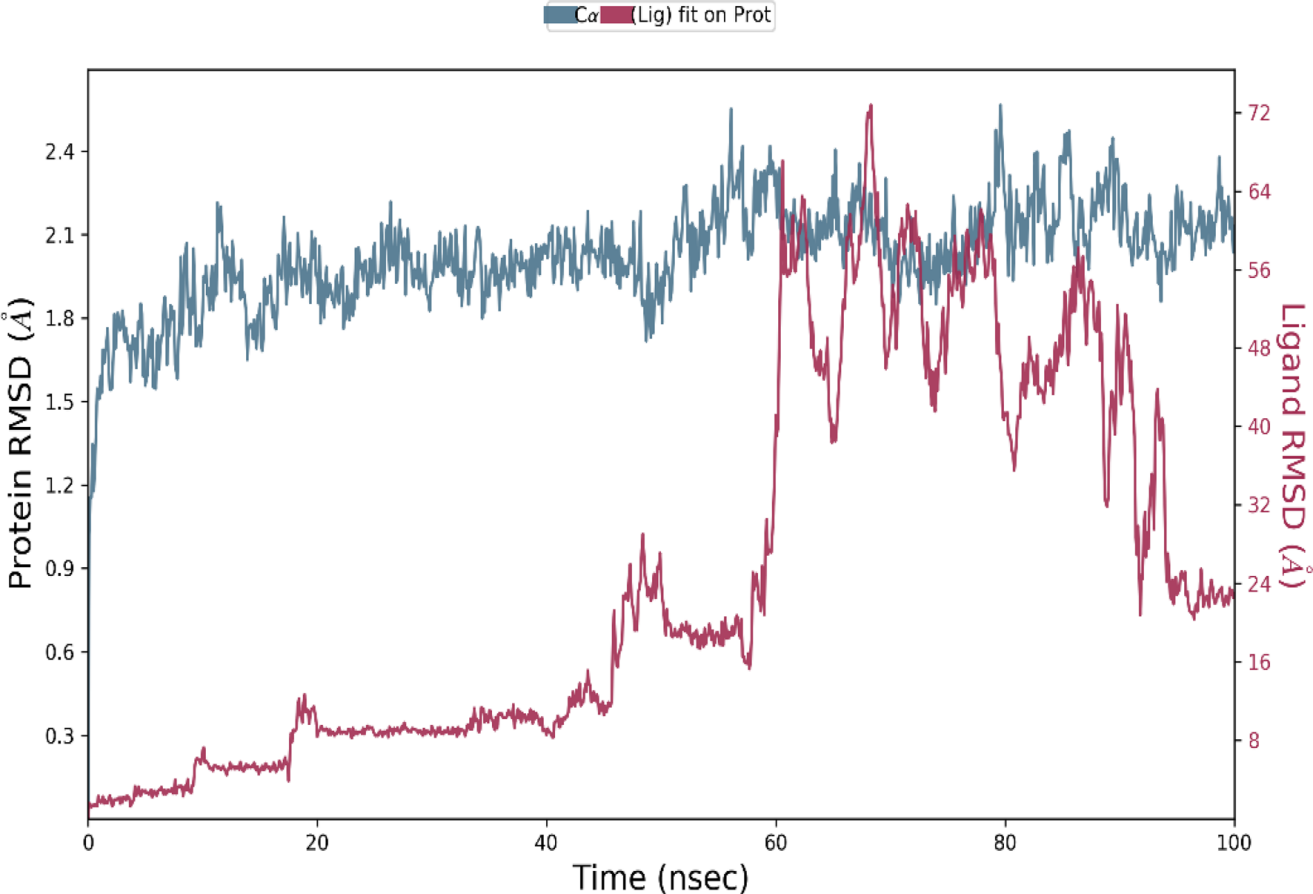
Protein–ligand RMSD Plot.

**Figure 8:**
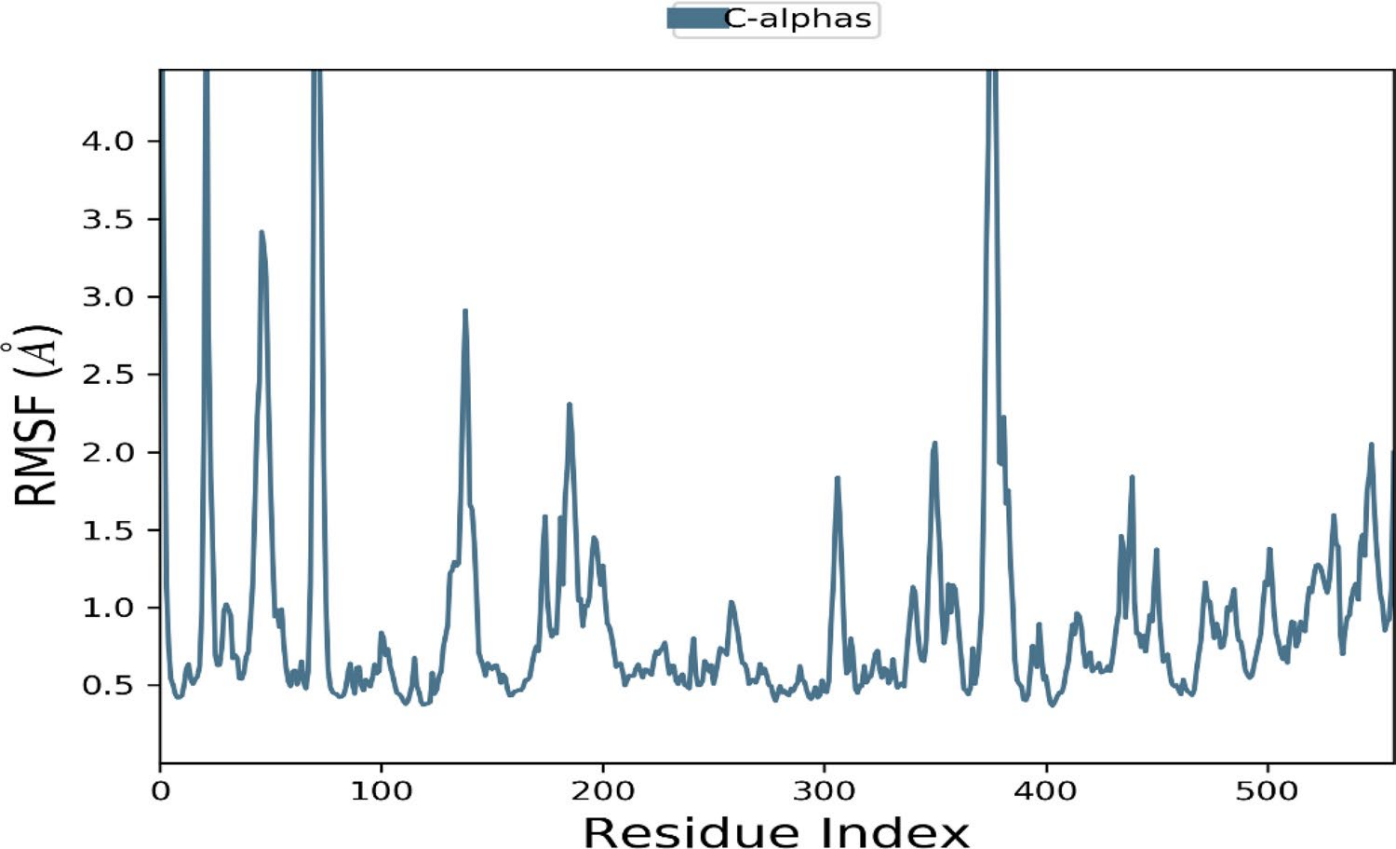
Protein RMSF Plot.

**Figure 9:**
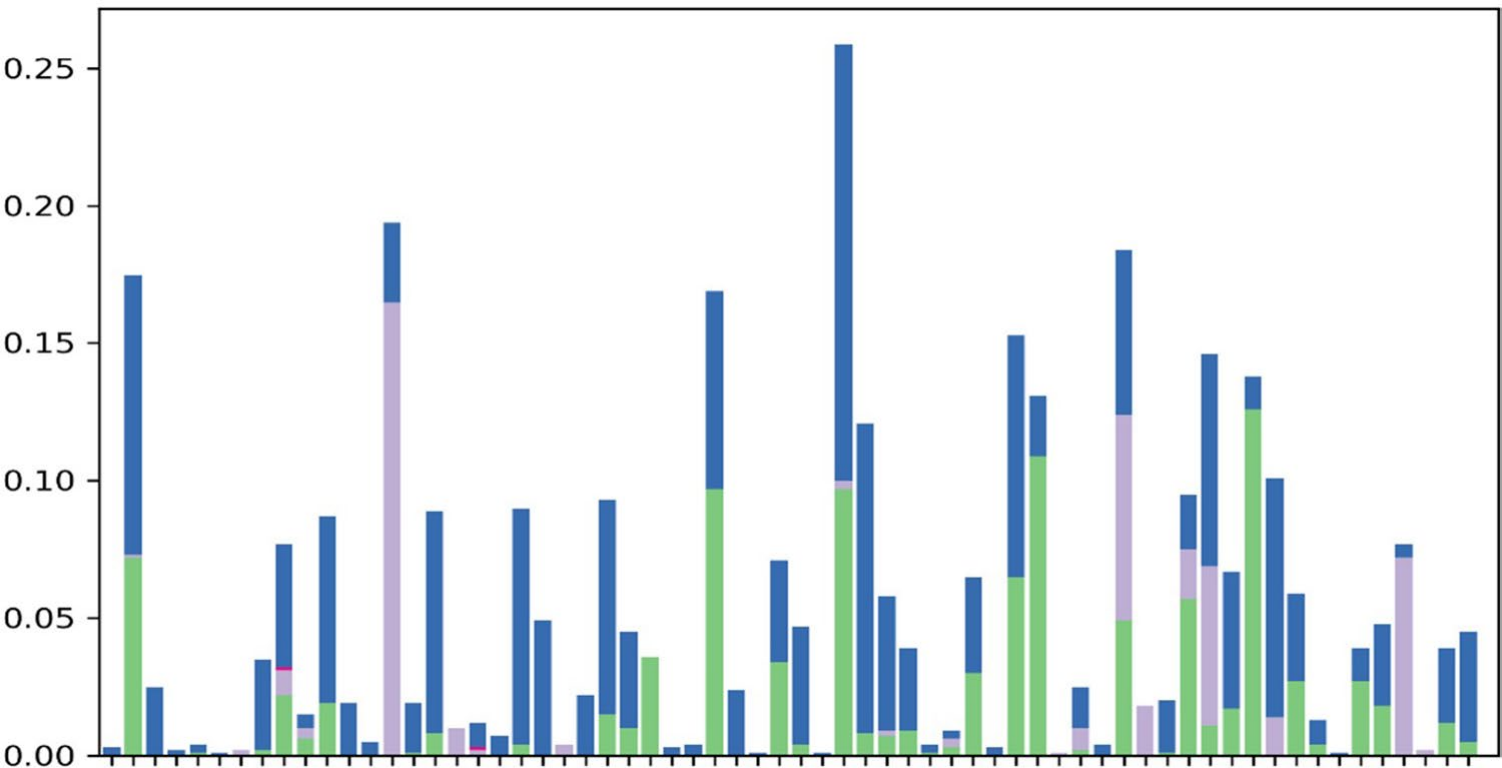
Protein–ligand contacts *(VAL 72, ARG 254, THR 257, LYS 259, THR 275, ALA 279, GLY 28, LYS 283, LYS 285, SER 337, LYS 340, ARG 372, TRP 374, ASN 377, GLY 378, LYS 380, ARG 380, HIS 354, SER 394, ASNB 395, PHE 397, GLY 400 , ASN 452, ASN 487, THR 488, ARG 490, ASP 492, PRO 521, VAL 522, TYR 523, GLY 524, TYR 525, MET 526, TYR 527, ASN 525, HIS 529, SER 531, TYR 531, LYS 532, SER 534, GLY 548, THR 549, GLY 550, VAL 552, LYS 583, THR 565, TYR 566, PHE 568, GLU 570, HIS 593, TYR 594, SER 595, GLY 596, ILE 597, GLN 598, GLU 599, ASN 602, GLN 608, GLN 632, PRO 633, PRO 635, ASN 677).*

**Figure 10:**
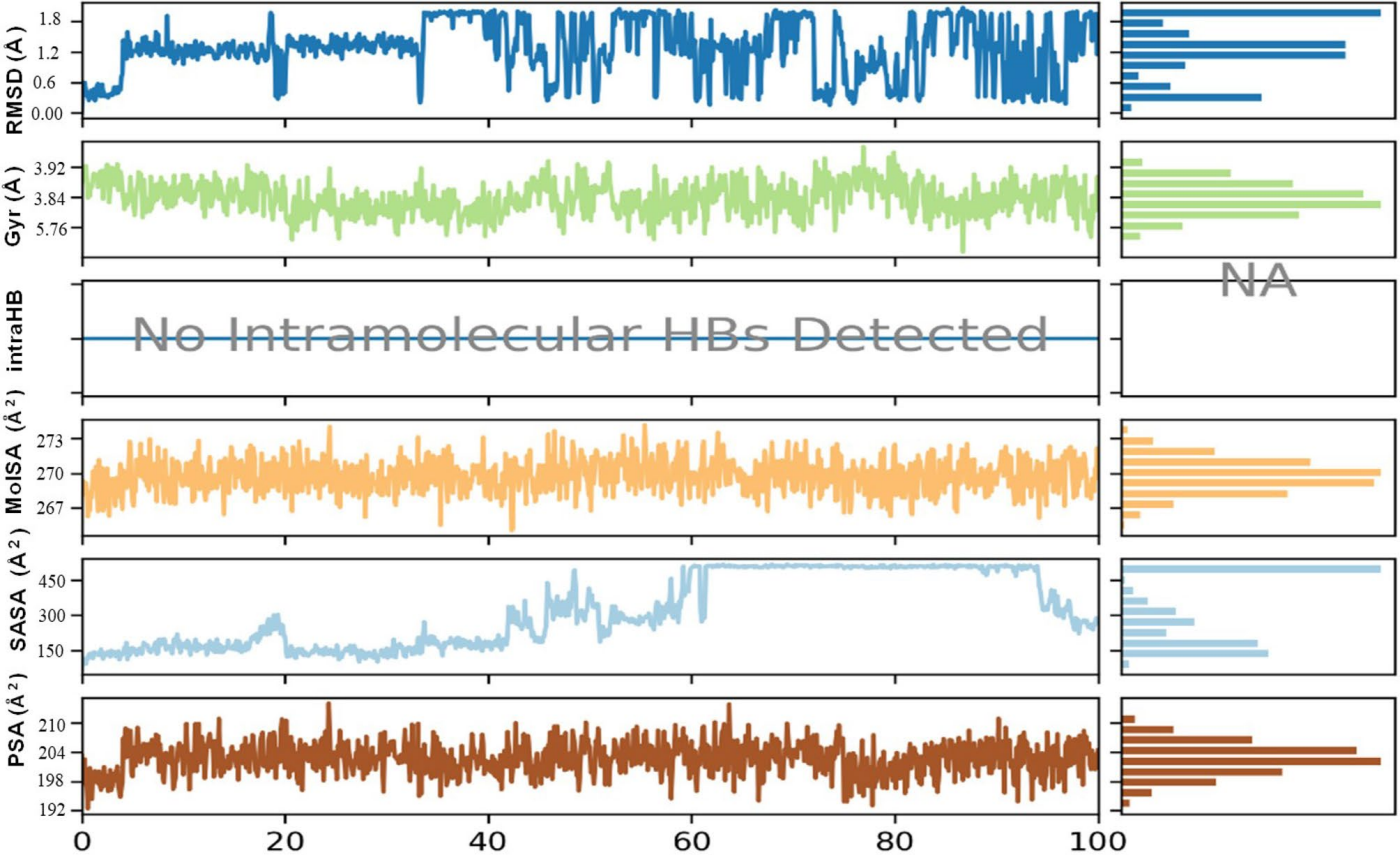
Root mean square deviation, radius of gyration, intramolecular hydrogen bonds, molecular surface area, solvent accessible surface area, polar surface area plots of the hit compound.

The best-hit compound was considered based on the binding affinity and transformation for molecular dynamic studies to analyze the stability of the compound in contrast to the protein of our interest. For simulation studies, the Desmond Schrodinger suite program was used for receptor complexes followed by the RMSD of protein exhibited a stable trajectory during the dynamic run for 100 nano seconds studies. Simulation of 100 nano seconds indicates significant conversions and stable conformations when comparing protein–ligand RMSD, as shown in Fig. 7. Regarding dynamic studies, RMSD exposed a fluctuated trajectory till 30 nano seconds, and there are fewer fluctuations between ligand and protein conformations (Fig. 7). Overall, both the receptor and ligand RMSD was observed in a stable format and can be seen in (Figs. 7, 8, 9, 10). In addition, all the amino acid interactions of our complexes were also analyzed accordingly, as well as the unique hydrogen bonds noted like TYR 561, ILE 371, ASN 397, and so on.

## Discussion

*F. cirrhosa* is a versatile medicinal plant with numerous biological properties in its nature. In the present study, the investigation of petroleum ether, ethyl acetate, and methanol extracts from bulbs of *F. cirrhosa* revealed the presence of various phytoconstituents, including flavonoids and carbohydrates saponins, phenols, alkaloids, steroids, and terpenoids. These bioactive phytoconstituents could be responsible for the therapeutic ability of various extracts of *F. cirrhosa*. The analysis was carried out by liquid chromatography-mass spectrometry (LC–MS), one of the most widely used techniques for separating phyto-constituents. The LC–MS investigation of *F. cirrhosa* bulb extracts revealed the presence of 88 phytochemical compounds, which could contribute to the medicinal properties of this plant species^14^. Solasodine (1S,2S,4S,5'R,6R,7S,8R,9S,12S,13R,16S)5',7,9,13tetramethylspiro[5oxapentacyclo[10.8.0.02,9.04,8.013,18]icos-18-ene-6,2'-piperidine]-16-ol) were extracted from *Solanum dulcamara* and evaluated for antimicrobial activity against the test bacteria like *Staphylococcus aureus*, *Enterobacter aerogenes*, *Escherichia coli*^45,46^. Linarin (5-hydroxy-2-(4-methoxyphenyl)-7-[(2S,3R,4S,5S,6R)-3,4,5-trihydroxy-6-[[(2R,3R,4R,5R,6S)-3,4,5-trihydroxy-6-methyloxan-2-yl]oxymethyl]oxan-2-yl]oxychromen-4-one) a bioactive flavone glycoside has been characterized abundantly in the *Cirsium*, *Micromeria*, and *Buddleja* species and exhibited promising activities particularly, the remedial effects on central nervous system (CNS) disorders^47^. Similarly, puerarin (7-hydroxy-3-(4-hydroxyphenyl)-8-[(2S,3R,4R,5S,6R)-3,4,5-trihydroxy-6-(hydroxymethyl)oxan-2-yl]chromen-4-one) is the major bioactive ingredient isolated from the root of the *Pueraria lobata* has been widely used in the treatment of cardiovascular and cerebrovascular diseases, diabetes and diabetic complications, osteonecrosis, Alzheimer’s disease, Parkinson’s disease, endometriosis, and cancer^48^. Likewise, Orientin isolated from different medicinal plants and possess promising pharmacological activities, which include antioxidant, antiaging, antiviral, antibacterial, anti-inflammation, vasodilatation and cardioprotective, radiation protective, neuroprotective, antidepressant-like, antiadipogenesis, and antinociceptive effects^49^. There are many reports on peonidin which exhibits apoptotic effects in the breast cancer cells and therapeutic applications^25,50^. Furthermore, alkaloids like sinpeinine A (17), imperialine-3-β-glucoside (18), imperialine (7) and 3β-acetylimperialine have been reported from Bulbus *F. cirrhosa*^14^. From the above evidence, it can be elucidated that *F. cirrhosa* consists of the enormous potential of pharmacological constituents therapeutic phytocompounds responsible for various pharmacological actions. This is the first report on LC–MS investigation of *F. cirrhosa* bulb extracts to the best of our knowledge. Furthermore, the present study investigated the in vitro antimicrobial activity against various bacterial and fungal strains. The study focused on the gram-positive bacteria and gram-negative bacteria, which shows the significant efficiency. Among various fungal and bacterial strains such as *E. coli, K. pneumonia, M. luteus, S. pneumonia, H. influenzae, N. mucosa and C. albicans, C. glabrata, C. Parapsilosis* were observed to be more susceptible to the plant extracts with the better minimum inhibitory concentration values (Table 4) compare to co- crystals MICs (0.625, 1.25, 0.039, 0.625, 1.25, 3.12, 1.25, 2 0.5, and 2.5) at a combinational concentration. In addition, it is also mentioned to that, the therapeutic herbs have different mechanisms that reflects identical antimicrobial mode of therapeutics in contrast to antimicrobial agents^10,51^. According to molecular docking, peonidin with 6 different proteins (dihydropterote synthase, penicillin binding protein, elongation factor Tu, 1,3 beta glycan, ABC transporter and beta-tubulin) exposed that the peonidin has a better interaction and binding score with penicillin binding protein as compared to other phytocompounds. The docking study results also showed that various energy sources are consistent and contribute to the overall strength of the binding interactions of peonidin for each target protein. After docking, computational molecular dynamic simulation studies revealed that various amino acids were observed to have interacted with peonidin, which is requisite for the synthesis of purines and pyrimidine nucleotides. Most of the interacted amino acids were bound with the hydrogen bonding and Vander Wall forces, whereas, the docking outcome with dihydropteroate synthase exposed the interrelated binding affinity with targeted receptors. The trajectory of molecular dynamic simulation was completed for 100 nano seconds and the RMSD and RMSF graph values were analyzed. The constant increase in the RMSD values with respect to time indicates that the protein serves from its native conformation regularly. The higher RMSF was analyzed in the study where strong hydrogen bonding was noted with mostly polar amino acids having maximum peak.

## Conclusion

The present investigation focused on identifying various bioactive compounds from the bulb extracts of *F. cirrhosa* for the first time by LC–MS analysis. These compounds are responsible for the different therapeutic and pharmacological properties. We have also provided evidence of bulb extracts of *F. cirrhosa* for its antimicrobial activity. Furthermore, the present study also explained the role of the peonidin compound against antimicrobial proteins such as dihydropteroate synthase (DHPS), penicillin-binding protein (PBP), elongation factor-Tu (Eu-Tu), 1,3 β-glycan, ABC transporter, and beta-tubulin. Among all the bacterial and fungal species, peonidin showed the best docking score against PBP and was then subjected to dynamic simulation studies to investigate the stability and RMSD and RMSF values concerning protein–ligand transformations. Using the peonidin compound may enable us to develop an effective drug against pathogenic bacteria and fungal diseases. In addition, ADMET (drug-likeness) studies showed the highest drug-likeness properties of the studied compound, which suggests that the peonidin compound can act as a promising microbail drug candidate. Further investigations to determine its bioactivity, toxicity profile, and clinical studies are necessary for broad-spectrum drug discovery.

## Supplementary Information

Supplementary Information.

## Abbreviations

ATCC: American Type Culture Collection Centre
CFU: Colony-forming units
CIP: Ciprofloxin
MIC: Minimum inhibitory concentration
MTCC: Microbial Type Culture Collection Centre
LC–MS: Liquid chromatography coupled with mass spectroscopy
UHPLC: Ultra-high-performance liquid chromatography
CBT: Centre for biodiversity and taxonomy
IMTECH: Institute of microbial technology
PBP: Penicillin binding protein
PAMPs: Pathogen associated molecular pattern
PDB: Protein Data Bank
RMSD: Root mean square deviation
RMSF: Root mean square fluctuation
MD: Molecular docking
MDS: Molecular dynamic simulation
